# Preoperative Nutritional Status and Its Consequences on Abdominal Surgery in Wolaita Zone, Southern Ethiopia: An Institution-Based Observational Study

**DOI:** 10.1155/2020/2324395

**Published:** 2020-06-09

**Authors:** Leila Hussen, Elazar Tadesse, Dereje Yohannes Teferi

**Affiliations:** ^1^School of Public Health, College of Health Science and Medicine Wolaita Sodo University, Wolaita Sodo, Ethiopia; ^2^Kotebe Metropolitan University, Addis Ababa, Ethiopia

## Abstract

This study aimed to determine the prevalence of malnutrition and its association with wound healing and length of hospitalization among patients undergoing abdominal surgery admitted to hospitals in the Wolaita zone in southern Ethiopia. *Methods*. An institution-based prospective observational study was conducted in three hospitals in the Wolaita zone from August to October 2016. All eligible individuals aged between 19 and 55 years were recruited in this study. Anthropometric and biochemical analyses, such as serum albumin (Alb) and total lymphocyte count (TLC), were taken for nutritional assessment during the preoperative period. Quantitative variables were compared using Student's *t* test. Cox's regression was employed to determine which variables were possible risk factors for poor wound healing. *Results*. A total of 105 patients aged 19 to 55 with a mean age (±SD) of 34 ± 9.6 years were included, and the prevalence of preoperative malnutrition was 27.6%, 87%, according to BMI and nutritional risk index, respectively. Poor wound healing was significantly associated with underweight patients (BMI < 18.5 kg/m^2^) (AHR: 6.5 : 95%CI: 3.312.9), postoperative weight loss (AHR: 4.9; 95%CI: 2.8–8.5), and nutritional risk index (NRI) less than 97.5 (AHR 1.8; 95% CI: 1.09–3.1). *Conclusion*. The prevalence of malnutrition is high in our study setup; this is associated with an increased risk of adverse postoperative outcomes. Therefore, our results emphasize the need of routine preoperative nutritional assessment, optimizing nutritional status of patients and postoperative nutritional support.

## 1. Introduction

Preoperative nutritional status has been shown to have important effects on health during recovery from illness or injury. Undernutrition develops as a result of scarcity in dietary intake, increased requirements associated with a disease state, complications of an underlying illness such as poor absorption and excessive nutrient losses, or a combination of these aforementioned factors. A positive outcome for surgery depends greatly on adequate immune defense and wound healing; it also requires a whole nutritional effort. Thus, the extent and time of healing of patients postoperatively significantly depends on the nutritional status of the individual [1–4].

Nutritional screening and therapy play an important role in the success of surgery; clean surgical wound often heal by primary intention. However, infection is one of the most common local factors that affect the healing process. Systematically, healing depends on the delivery of blood with the delivery of oxygen, nutrients, and leukocytes to the wound site. Some specific conditions like anemia and systemic disease can weaken the healing process [5–7].

Patients who undergo abdominal surgery are at risk of malnutrition due to periods of prolonged starvation time, stress of surgery, and subsequent increase in metabolic rate [8, 9].

Undernutrition is a major public health problem that is underrecognized, undertreated, worsens in hospitals, and common throughout in the world. The nutritional status of a patient during hospital admission influences the outcome of the patient, and nutritional intervention during hospital stay improves the outcomes of surgery [10].

Despite the current recommendations stipulating screening for the nutritional status and risk stratification for all patients in the preoperative period, nutritional screening and therapy are not an integral part of daily clinical practice in many of the hospitals in Ethiopia, irrespective of the nutritional status all the patients are provided with the same diet from the central cafeteria. In Ethiopia, there are limited data regarding the prevalence and effects of preoperative nutritional status on wound healing and length of stay among patients undergoing abdominal surgery. Therefore, this study was designed to investigate this problem.

## 2. Materials and Methods

### 2.1. Study Design and Setting

This study was an institutional-based prospective observational study conducted in three hospitals in Wolaita zone, southern Ethiopia, namely, Wolaita Soddo Referral Hospital (governmental), Soddo Christian Hospital (private), and Dubbo Hospital (Private). Wolaita is one of the fourteen zones of South Nation, Nationalities, and People Region (SNNPR). It is located 329 km southwest of Addis Ababa and 167 km south of the regional capital city Hawassa. The population of soddo is estimated to be 100,000 and 1.7 million in the Wolaita zone as general.

### 2.2. Study Participants

This study included all consecutive eligible abdominal surgery patients aged between 19 and 55 years, and data collection was carried out from August to October 2016. At the preoperative period with informed consent, anthropometric, biochemical, and clinical data were carried out on admission, and follow-up took place on the 3^rd^ and 5^th^ postoperative days and discharge. Patients with malignancies, whose nutritional status was not determined during the preoperative period, were excluded.

### 2.3. Data Collection and Measurements

The data regarding demographic variables like age, sex, and socioeconomic status were collected using structured interviewer-administered questionnaires. Medical records were reviewed to collect clinical data such as past medical and surgical history and gastrointestinal symptoms.

Weight was measured on admission and at discharge with light clothing to the nearest 0.1 kg with a digital Seca scale balance and height to the nearest 0.1 cm using a portable calibrated stadiometer.

 
*Ideal bodyweight* was calculated according to the Lorentz formula that takes into account the patient's height and sex as follows: IBW (kg) for men = height (cm) − 100 − {[height (cm) − 150]/4} [11]; IBW (kg) for women = height (cm) − 100 − {[height (cm) − 150]/2} [12].
 
*Body mass index (BMI)* was computed as body weight (kg) divided by height squared (m). Patients were classified into WHO categories. The nutritional status is defined by cutoff points of as follows: underweight (<18.5 kg/m^2^), normal range (18.5–24.9 kg/m^2^), overweight (25–29.9 kg/m^2^), and obesity (≥30 kg/m^2^) [13].
 
*Nutritional risk index (NRI)* was originally derived from the serum albumin concentration and the ratio of the present to the usual weight. Encountered with difficulty to find the usual body weight of patients, we used ideal body weight instead of usual body weight in the NRI formula as follows: NRI = (1.519 × serum albumins, g/dl) + {41.7 × present weight (kg)/ideal body weight (kg)}. Using this index, all patients were classified into two categories, namely, not malnourished (NRI ≥ 97.5) and malnourished (NRI < 97.5). Malnutrition was also ranked as moderate between 83.5 and 97.5 and severe (NRI < 83.5). For analysis, the patients were stratified into two groups as follows: malnourished NRI < 97.5 and not malnourished NRI ≥ 97.5 [11, 14].

### 2.4. Biochemical Measurement

Blood samples for albumin and total lymphocyte count were collected from the peripheral vein using plain and blood collection systems and stainless steel needles. Serum albumin levels were measured by the bromocresol green colorimetric method. The degree of malnutrition was defined by cutoff points, serum albumin ≥3.5 g/dl (normal), based on total lymphocyte count (cells/mm^3^): >1500 (normal) and <1500 (malnourished) [15].

### 2.5. Assessment of Wound Complications

The postoperative wound was assessed using a wound healing checklist, derived from Oh et al. [11]. Complications that developed during the hospital stay and the types of wound complications were classified as follows:

  Seroma: serous fluid collection in the absence of infection under the surface of the skin
  Hematoma: subcutaneous blood in the absence of infection
  Wound infection: two or more of the following manifestation drainage of purulent discharges: redness, increase pain, indurations, and fever
  Wound dehiscence: hematoma seroma or infection that required the incision to be opened or evacuated, irrigated, and debrided, a status required to be healed by secondary intention
  Length of hospital stay (LOS): computed from the day of surgery to hospital discharge

### 2.6. Data Management Analysis Procedures

Data were entered into Epi-Info (Version 6.0), cleaned, and exported to IBM SPSS for Windows (Version 20.0) for analysis. The percentage was used for categorical variables, and differences in proportions were compared using the chi-squared test. Mean ± standard deviation (SD) was used for the continuous variable. Length of stay between the patient group (undernourished versus normal) was evaluated using Student's *t* test. A *p* value of <0.05 was considered significant. Cox regression bivariate analysis was done, and variables with a *p* value of <0.25 entered the multivariable Cox regression. Finally, multivariable Cox regression analysis was done to control the potential confounders and identify independent predictors of the outcome. Their hazard risk was computed with their CI; a *p* value of <0.05 was considered significant.

## 3. Results

**Figure 1:**
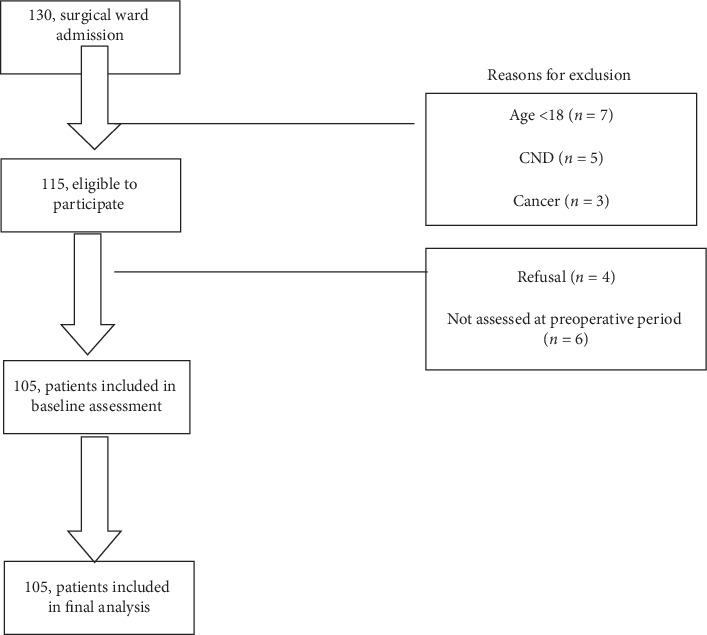
Study participant's flow chart in hospitals at Wolaita zone, southern Ethiopia, 2016. CND: chronic noncommunicable disease.

Of the 115 eligible admissions followed, 105 (91.3%) were included in the ultimate analysis (Figure 1).

### 3.1. General Characteristics of the Respondents

A total of 105 respondents were studied. The mean age ± SD was 34 (±9.6) years, and almost half of the respondents 50 (47.6%) were between the ages of 25–40, with 52% of female and about one-third of the respondents having no formal education 31 (29.5%). Most of the patients (78 (74.3%)) were married, and 59 (56.2%) had a monthly income of less than 15 USD. The main reason for admission was gastrointestinal surgery (55.2%) (Table 1).

### 3.2. Pre- and Postoperative Nutritional Statuses

Before surgery, the BMI of respondents ranged from 14 to 30 kg/m^2^ and mean BMI (±SD) was 21.3 (±3.77) kg/m^2^. About a third, 29 (28.6%) patients, had a BMI of less than 18.5 kg/m^2^ (undernutrition), of which 9 (8.6%) had a BMI less than 16 kg/m^2^ (severe undernutrition) and 5 (4.8%) and 15 (14.3%) moderate to mild undernutrition, respectively. About two-thirds, 60 (57.1%), were between 18.5 and 24.9 kg/m^2^ (normal), and the remaining 14.3% (15) were between 25 and 29.9 kg/m^2^ (over weight). On the other hand, low serum albumin levels (<3.5 g/dL) were observed in 67.6% (*n* = 71/105) participants.

According to their total lymphocyte count, 29 (27.6%) had low count (<1500 cells/mm^3^). Using the nutritional risk index (NRI), 20.9% had no risk. The remaining 37% (*n* = 39/100) and 37% (*n* = 39/100) each were at moderate and severe risk, respectively.

After surgery, the BMI of respondents ranged from 13 to 30 kg/m^2^ and mean BMI (±SD) was 20.6 (±4) kg/m^2^. 33 (32.4%) patients had undernutrition (BMI less than 18.5 kg/m^2^). Similarly, the proportion of moderate and severe undernutrition increased at the postoperative period (16 (15.2%) and 7 (6.7)), respectively (Table 2).

### 3.3. Postoperative Outcomes

Postoperative wound complications occurred in 21% (22/105) of respondents; comparing wound complications with nutritional status, 86.4% (19/22) belonged to the malnourished group according to BMI and 95.5% (21/22) belonged to the malnourished group according to NRI. During the hospital stay, the mean weight loss was 1.34 ± 2.23 kg, most (65%) of the patients presented with weight loss, 18.3% gained weight, and the remaining 16.2% had no change during their stay; a total 95.2% (20/21) patients with intrahospital weight loss experienced wound complications (Table 3).

### 3.4. Effect of Nutritional Status on Length of Stay

The mean length of stay was 6 ± 2.6 days; longer hospital stay was associated with preoperative nutritional status and intrahospital weight loss. According to NRI, malnourished patients stayed longer than not malnourished patients (7 ± 2 days versus 4 ± 1 days, *p* < 0.001). The mean LOS for malnourished patients using BMI was longer (9 ± 2 days versus 5 ± 1 days, *p* < 0.001).

**Figure 2:**
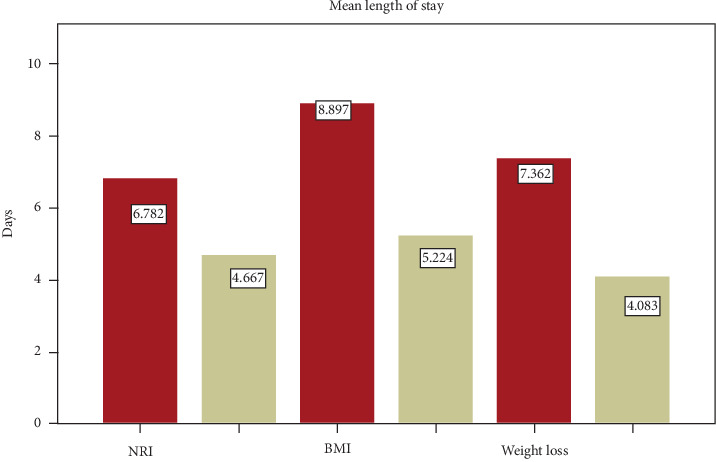
Comparison of length of stay in patients undergoing abdominal surgery with normal nutritional status (gray bar) and underweight (red bar); patients with postoperative weight loss (red bar); and no change or gain weight (gray bar) among those undergoing abdominal surgery in hospitals at Wolaita zone in southern Ethiopia, October 2016. NRI: nutritional risk index; BMI: body mass index; weight loss: postoperative weight loss.

Most (65%) of the patients presented with weight loss of 18.3% gained weight; the remaining 16.2% had no change during their stay. On the other hand, intrahospital weight loss was associated with length of hospital stay; the mean LOS for patients who had weight loss was 7 ± 2 days and 4 ± 1 days, for those who gained weight or had no change (Figure 2).

### 3.5. Factors Associated with Poor Wound Heal1ing Status

In this study, BMI at admission was significantly associated with wound healing complications. Underweight patients were about six times more likely to have poor wound healing (AHR: 6.2; 95% CI: 3.1, 12.5).

Patients who had a nutritional risk index (NRI) less than 97 (malnourished) were two times more likely to have wound healing complications compared with those who had NRI 97 and above (not malnourished) (AHR: 1.8; 95% CI: 1.09, 3.1).

The risk of getting poor wound healing was higher among patients who had lost weight in the postoperative period compared with patients with weight gain or no change (AHR: 4.9; 95% CI: 2.8, 8.5) (Table 4).

## 4. Discussion

Malnutrition is a significant risk factor for postoperative complications in major abdominal surgery. In the present study, a high occurrence of malnutrition was found in the study population. One-fourth of the participants developed wound complications.

The nutritional parameters associated with poor wound healing were as follows: body mass index at admission (BMI < 18 kg/m^2^), preoperative nutritional risk index (NRI < 97), and postoperative weight loss. The prevalence of malnutrition according to BMI was 27.6%. We observed a higher proportion of undernutrition in the current study as compared with similar studies in sub-Saharan Africa (16%) and slightly less than the similar study findings from India (36%) [14, 16].

The prevalence of malnutrition according to the nutritional risk index (NRI) was 74%, which is superior to the reported prevalence in sub-Saharan Africa (39.1%) possibly because their study sample included a higher proportion of minor abdominal surgical procedures known to have short pre- and postoperative fasting times (14). Patients who undergo abdominal surgery are unsurprisingly at greatest risk of malnutrition due to long periods of starvation before, and after surgery, the stress of surgery and an increase in metabolic rate after surgery [10, 11]. In this study, the prevalence of malnutrition increased in the postoperative period (27.6%–32.4%). This finding is supported by a systematic review article which was conducted in Latin America [6].

Nutrition is one of the most important factors affecting wound healing. Together with fats, carbohydrates are the primary source of energy in the wound-healing process. A deficiency of carbohydrates and proteins can impair capillary formation, fibroblast proliferation, collagen synthesis, and wound remodeling. A deficiency of protein also affects the immune system and increases susceptibility to infection. Stress following surgery increases the need for energy and nutrients at a time when food intake is frequently reduced [9, 17]. The BMI of patients with digestive tract diseases was a very good indicator of nutritional status, and the relative risk of complications doubles in malnourished patients. In this study, 86.4% of underweight patients developed wound healing complication. The finding of the study also revealed that underweight individuals were about 6 times more likely to have poor wound healing status, besides requiring significant longer stay than nourished patients (9 ± 2 versus 5 ± 1 days). Like the present study, another study also found that unfavorable surgical outcomes, including problems with wound dehiscence, correlated well with preoperative undernutrition (18). On the other hand, it was reported that the preoperative nutritional risk index was strongly associated with postoperative outcome (14). In the present study, it was found that 95.5% of malnourished patients developed wound healing complications and also 2 times more likely to have wound healing complications and require a significantly longer stay than well-nourished patients (7 ± 2 versus4 ± 1 days). This finding is supported by different literatures [6, 14, 18, 19].

In this study, we found that weight loss during hospitalization was associated with poor wound healing and prolonged length of stay; the study performed in the USA revealed that decline in nutritional status was associated with a greater risk of complication [20]. Likewise the study patients who lose their weight during hospital stay were about 5 times more likely to have wound healing complication and stay long in hospitals (7 ± 2 VS4 ± 1 days). This finding was supported by different literatures [6, 10, 21].

This study has the following limitations. The dietary assessment was not addressed, and the study considered a small sample size. The daily menu of the patients was not well assessed, so that this study was unable to associate the hospital's daily feeding menu with the intrahospital weight loss of the respondents. So, we recommend further study, involving the hospital's feeding protocol of admitted patients.

## 5. Conclusion

This study suggests that preoperative poor nutritional status is an important independent predictor of poor wound healing and longer hospital stay. In this study, we found that a high prevalence of malnutrition further exacerbated in the postoperative period. To ensure a good outcome, it is highly recommended to determine the nutritional status of patients at admissions. Besides, individualized nutritional supplementation and nutritional education must be in place.

## Figures and Tables

**Table 1:** General characteristics of respondents at hospitals in Wolaita zone, Southern Ethiopia, October 2016.

Characteristics (*n* = 105)	*N* (%)

Age of respondents (year)	
19–24	28 (26.7)
25–40	50 (27.6)
41–55	27 (25.7)

Job	
Merchant	28 (26.7)
Government employees	15 (14.2)
Farmer	41 (39)
Student	7 (6.7)
No job	7 (6.7)
Others^*∗*^	7 (6.7)

Sex	
Male	50 (47.6)
Female	55 (52.4)

Marital status	
Married	78 (74. 3)
Never married	22 (21)
Divorced/widowed	5 (1.8)

Level of education	
Illiterate	31 (29.5)
Primary school	25 (23.8)
Secondary school	14 (13.5)
High school	16 (15.2)
College and above	19 (18.1)

Religion	
Christian	92 (87.6)
Muslim	13 (12.4)

Ethnicity	
Wolaita	53 (50.5)
Hadiya	6 (5.7)
Kenbata	10 (9.5)
Oromo	11 (10.5)
Gamo Gofa	13 (12.4)
Others	12 (11.4)

Monthly income (USD)	
<15	59 (56.2)
15–34	16 (16.2)
33–88	18 (13.3)
>88	12 (11.4)

USD :United States Dollar. ^*∗*^Others include daily laborers and nongovernmental organizations.

**Table 2:** Distribution types of wound healing complication according to different parameters in Wolaita zone, Southern Ethiopia, October 2016.

	NRI		BMI (kg/m^2^)		Weight loss during hospital stay	
	<97.5	≥97.5	<18.5	≥18.5	Loss	Gain/no change
Seroma	6	0	5	1	6	0
Hematoma	8	0	7	1	7	1
Wound infection	5	0	4	1	5	0
Wound dehiscence	2	1	3	0	3	0
Total	21	1	19	3	21	1

NRI: nutritional risk index. BMI: body mass index.

**Table 3:** Pre- and postoperative nutritional status of respondents according to different parameters in Wolaita zone, southern Ethiopia, October 2016.

	*N* (%)
*Preoperative nutritional status*	
BMI (kg/m^2^)	
Mean (±SD)	21.3 (±3.77)

<16 kg/m^2^	9 (8.6)
16–16.9	5 (4.8)
17–18.4	15 (4.3)
18.5–24.9	60 (57.1)
25–29.9	15 (14.3)
LSA (g/dl)	
Mean (±SD)	3.1 (0.83)
≤3.5	71 (67)
>3.5	34 (33)
TLC (mm^3^)	
Mean (±SD)	1928 (707)
<1500	29 (27.6)
≥1500	76 (79.4)
NRI	
≥97.5	22 (20.9)
83.5<97.5	39 (37.1)
<83.5	39 (37.1)

*Postoperative nutritional status*	
BMI (kg/m^2^)	
Mean (±SD)	20.6 (±4) kg/m^2^
<16 kg/m^2^	16 (15.2)
16–16.9	7 (6.7)
17–18.4	11 (10.5)
18.5–24.9	58 (55.2)
25–29.9	13 (12.4)

BMI: body mass index. LSA: level of serum albumin. TLC: total lymphocyte count. NRI: nutritional risk index.

**Table 4:** Factors associated with poor wound healing status among patients undergoing abdominal surgery in hospitals at Wolaita zone in southern Ethiopia, October 2016.

Variables		Wound healed		CHR (95%CI)	*p* value	AHR (95%CI)	*p* value
		Yes (%)	No (%)				
LSA (g/dl)	≤3.5	53 (63.9)	18 (81.1)	1.72 (2.3–3.1)	0.02	1.5 (0.231–2.1)	0.712
	>3.5	30 (36.1)	4 (18.2)	1			
TLC (cells/mm^3^)	<1500	20 (24.1)	63 (82.9)	1.6 (1.02–2.7)	0.04	1.9 (0.85–42)	0.174
	≥1500	63 (75.9)	13 (17.1)	1		1	
BMI (kg/m^2^)	<18.5	10 (12)	19 (86.4)	6.5 (3.3–12.1)	<0.001	6 (3.1–12)	<0.001^*∗∗∗*^
	≥18.5	73 (88)	3 (13.6)	1		1	
Weight in hospital stay	Weight loss	48 (57.8)	21 (95.5)	3.1 (2.3, 4.3)	<0.001	3 (2.6–7.4)	<0.001^*∗∗∗*^
	Weight gain/no change	35 (42.2)	1 (4.5)	1		1	
NRI	<97.5	57 (68.7)	21 (95.5)	2.2 (1.3–3.81)	0.01	2.1 (1.2–3.6)	0.003^*∗∗∗*^
	≥97.5	26 (31.3)	1 (4.5)	1		1	

## Data Availability

All results of this research were based on the use of primary data, and the data collection was performed prospectively. The datasets used during the current study are available from the corresponding author upon reasonable request.
